# Supplementary material for: Bacteria-on-a-bead: probing the hydrodynamic interplay of dynamic cell appendages during cell separation
Source: Commun Biol. 2022 Oct 14;5:1093. doi: 10.1038/s42003-022-04026-z (PMC9568603; doi:10.1038/s42003-022-04026-z)

Supplementary Information to

Bacteria-on-a-Bead: Probing the Hydrodynamic Interplay of Dynamic Cell Appendages during Cell Separation

---

Nora Sauter[1,2,3,+,‖], Matteo Sangermani[4,±,‖], Isabelle Hug[4], Urs Jenal[4], and Thomas Pfohl[1,2,3]*

[1] Institute of Physics, University of Freiburg, Hermann-Herder-Str. 3, 79104 Freiburg, Germany.

[2] Department of Chemistry, University of Basel, Klingelbergstrasse 80, 4056 Basel, Switzerland.

[3] Swiss Nanoscience Institute, 4056 Basel, Switzerland

[4] Biozentrum, University of Basel, Klingelbergstrasse 50/70, 4056 Basel, Switzerland.

[+] Present address: Bundesamt für Landwirtschaft, Eidgenössisches Departement für Wirtschaft, Bildung und Forschung, Schwarzenburgstrasse 165, 3003 Bern, Switzerland.

[±] Present address: Department of Circulation and Medical Imaging, Norwegian University of Science and Technology, Olav Kyrres gate 9, 7030 Trondheim, Norway.

[‖] These authors contributed equally

* Correspondence: thomas.pfohl@physik.uni-freiburg.de

Supplementary Note 1: Composition of peptone yeast extract (PYE)

For 1 l

Bacto Peptone: 2 g (BD, Art. Nr. 211677)

Yeast Extract: 1 g (Oxoid, Art. Nr. L21)

$MgSO_4$ 0.5 M: 1.6 ml ($MgSO_4$ x 7 H_2O, Merck, Art. Nr, 1.05886.0500)

Dest. H_2O: 1 l

The ingredients were dissolved in water, poured into smaller glass bottles and autoclaved. After autoclaving the bottles were stored at room temperature. Before use 0.5 mM $CaCl_2$ was added.

Supplementary Figure 1

Surface attachment efficiency of bacteria-on-a-bead.

a: Normalized numbers of attached cells per bead for the differently functionalized beads (*N* > 9 per measurement, at least two different measurements per time point and functionalization, error bars represent ± standard deviation (SD). Diameter of the bead: 3 µm).

b: The attachment of the cells on a bead depends on the size and the surface coating of the bead. Micrographs of attached cells on amino- and carboxy-coated beads with a diameter of 3 µm (top) and 1 µm (bottom).

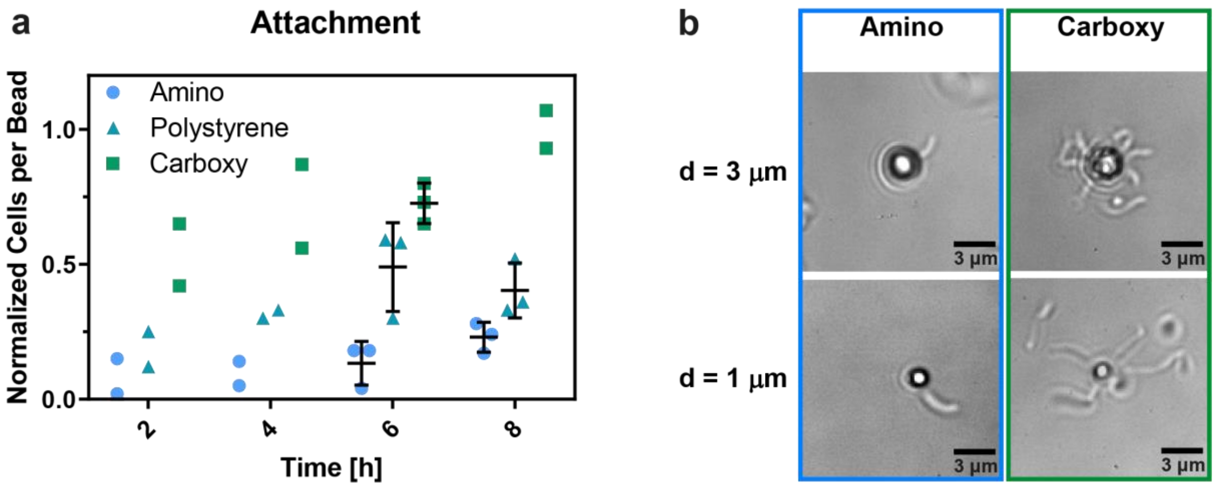

Supplementary Figure 2

Characterization of predivisional cells with flagellar activity attached to small beads.

Flagellar activity before cell separation; the overall activity (*N* = 10, error bars represent ± SD) from the firstly observed swimming until cell separation (left) and the time of the rotation daughter side before separation from the mother side (*N* = 3, error bars represent ± SD).

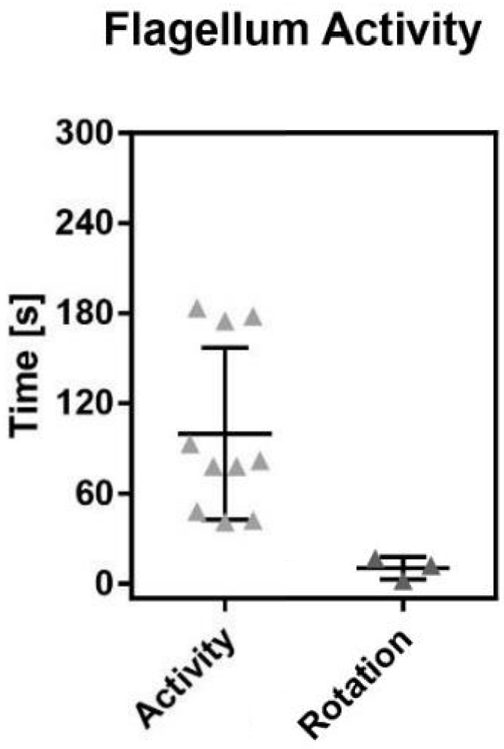

Supplementary Figure 3

Bead and cell position of a cell without a flagellum (Δ*flgDE*) in optical tweezers: Cell separation is indicated by a dashed line.

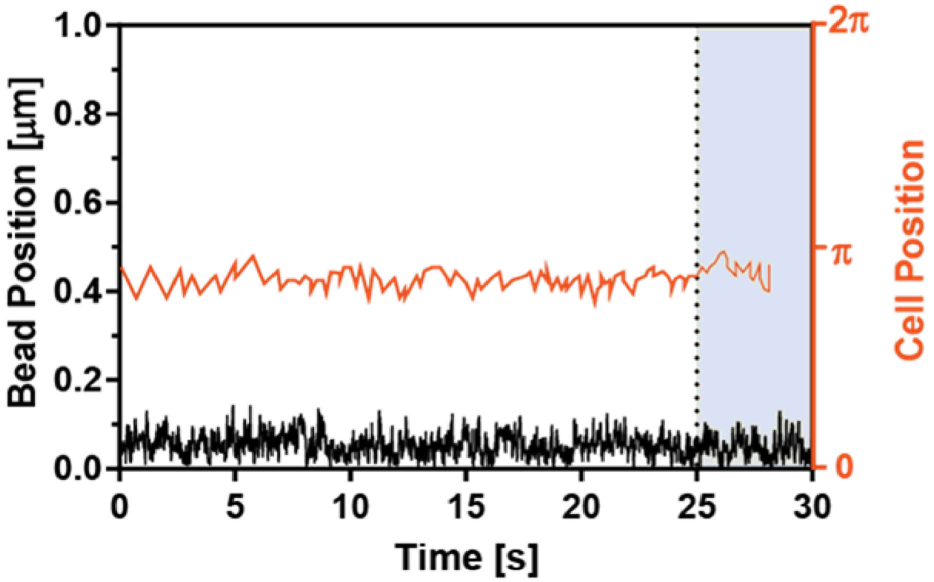

Supplementary Figure 4

Characterization of predivisional cells with active flagellum attached to beads in optical tweezers.

The overall activity from first observed gyrational movement until cell separation is divided into two stages, the gyrational stage, where the predivisional cell shows a movement of the two-cell side together and a rotation stage, where the daughter side rotates around its own axis (*N* = 8 for activity and movement, *N* = 10 for rotation, error bars represent ± SD).

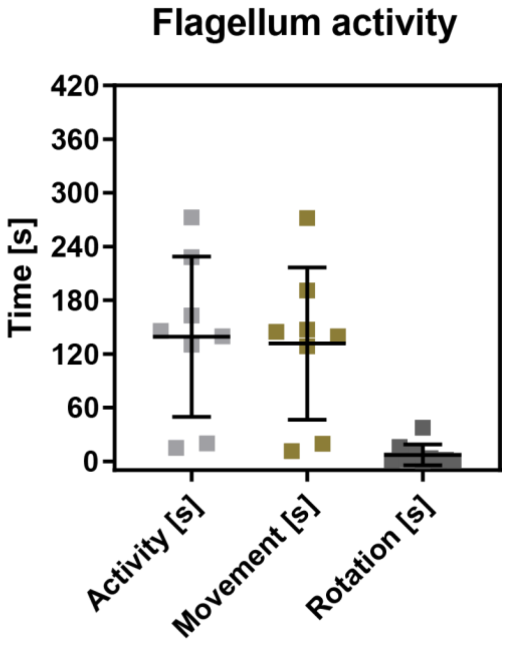

Supplementary Figure 5

Characterization of the rotating stage of the daughter cells.

a: Normalized fraction of cells that start the rotation of the predivisional daughter cell in CCW, resp. CW direction (N = 12, error bars represent ± $\sqrt{(\text{number of observed events})}$).

b: Duration of the CCW and CW sequences of the predivisional daughter cell (N = 10 individual cells, 17 observed sequences in CCW and N = 7 individual cells, 12 observed sequences in CW, error bars represent ± SD).

c: Rotation frequency of sequences in CCW, respectively CW direction (CCW: N = 11, CW: N = 6, error bars represent ± SD).

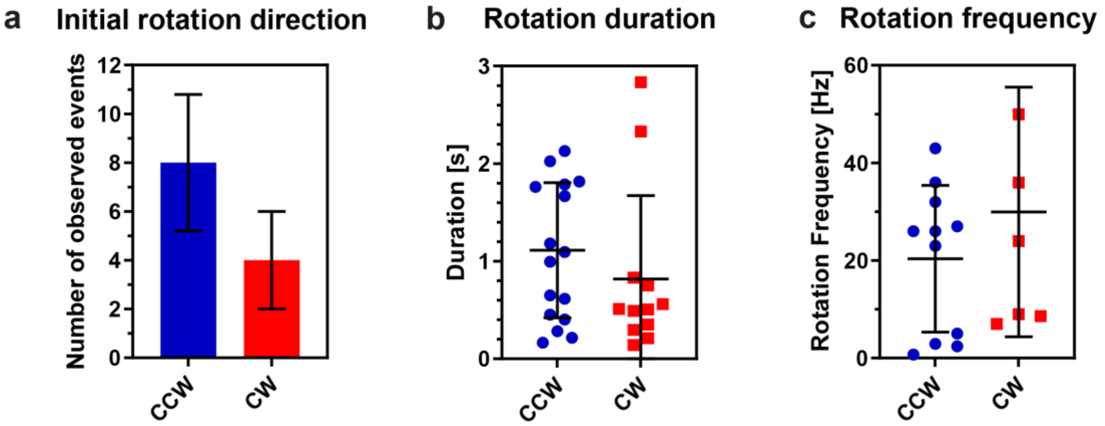

Supplementary Figure 6

Schematic representation and dimensions of the setup for determining the interplay and competition of flagellum and pili.

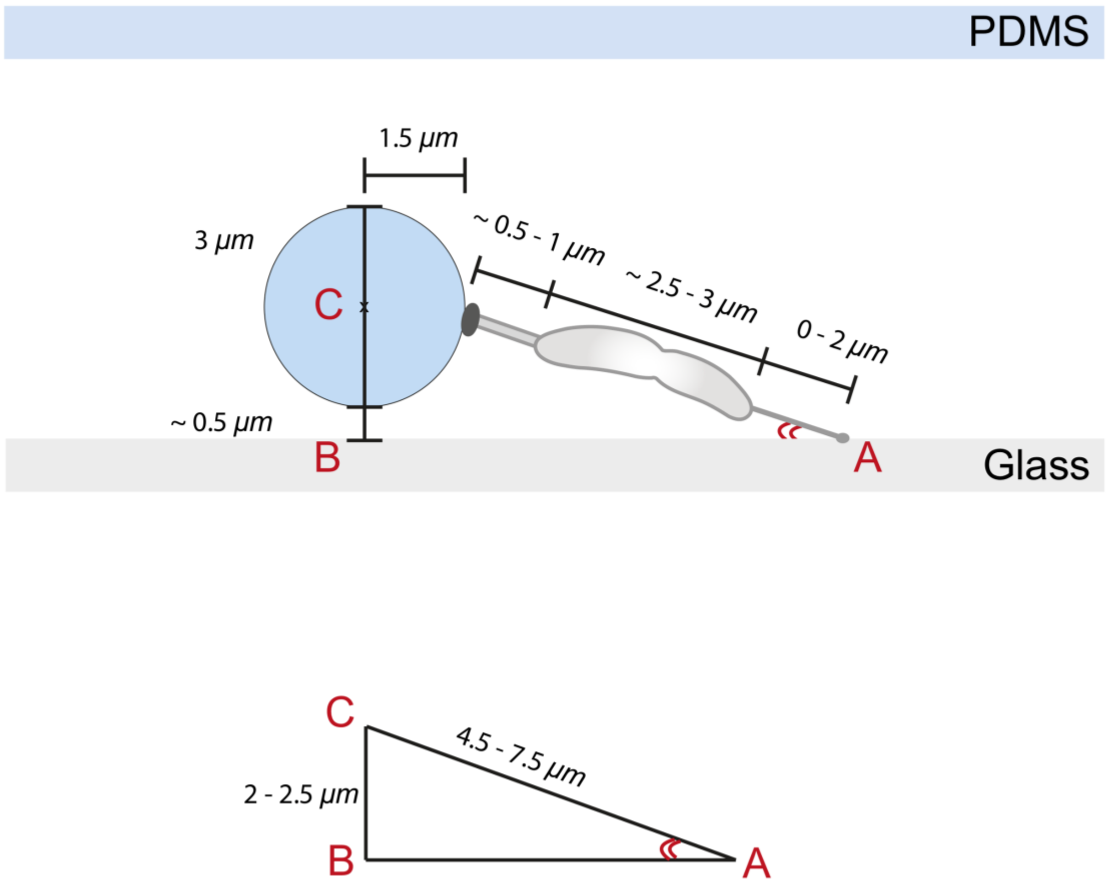

Supplementary Figure 7

Competition between flagellum and pili.

a: Individual duration of observed movement for WT that is caused by pili or flagellum respectively (*N* = 9, error bars represent ± SD).

b: Period in between individual pilus attachment cycles for Δ*flgDE* (*N* = 25)

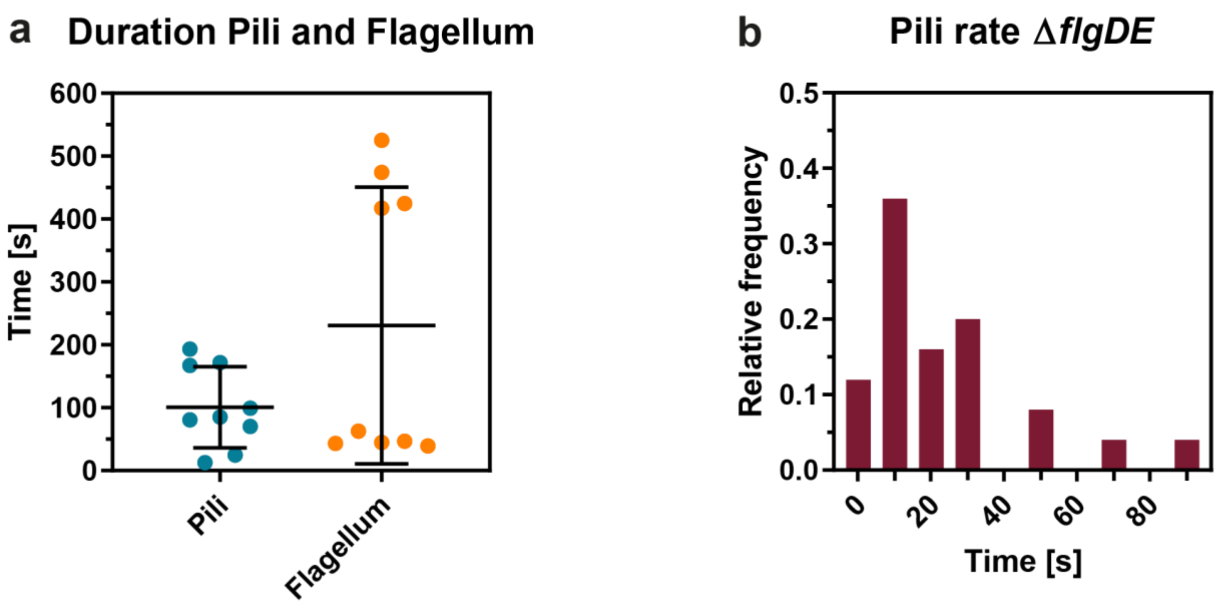

Supplement: Supplementary file 2 — Supplementary Information [file 42003_2022_4026_MOESM2_ESM.pdf]
