# Supplementary material for: Bacteria-on-a-bead: probing the hydrodynamic interplay of dynamic cell appendages during cell separation
Source: Commun Biol. 2022 Oct 14;5:1093. doi: 10.1038/s42003-022-04026-z (PMC9568603; doi:10.1038/s42003-022-04026-z)
Supplement: Supplementary file 3 — Description of Additional Supplementary Files [file 42003_2022_4026_MOESM3_ESM.pdf]

Description of Additional Supplementary Files

File name: Supplementary Movie 1

Description: Swimming predivisional WT cells attached to a polystyrene bead with diameter 1 µm in PYE with overlaid track

File name: Supplementary Movie 2

Description: Gyrational motion of predivisional WT cells attached to a bead with diameter 3 µm in PYE in optical tweezers: Mother and the daughter side are still firmly connected; the rotation of the flagellum induces a gyrational motion of the bead and the whole cell around the center of the optical tweezers.

File name: Supplementary Movie 3

Description: Gyrational motion of predivisional NA1000 ΔcheYII hsfA+ (this work). A clean deletion of CC0437 causes the flagellum to not change its rotation direction, it keeps rotation in CW direction (CCW rotation direction of cell body). Bacteria-on-a-bead system, consisting of a predivisional cells attached to a bead with diameter 3 µm in PYE in optical tweezers. The connection between mother and daughter side is softened, the rotation of the flagellum causes a rotation of the daughter side around its long axis. The gyration of the predivisional cell proceeds but is slowed down.

File name: Supplementary Movie 4

Description: Three 'motile' stages of predivisional WT cells attached to a glass substrate ("infinite counter mass") in PYE. Bending, rotation and cell segregation with swarmer cell release.

File name: Supplementary Data 1

Description: The source data behind the graphs in the main text.

File name: Supplementary Data 2

Description: The source data behind the graphs in the Supplementary Information.
